# Effect of sheep placenta extract on D-galactose-induced aging mouse

**DOI:** 10.3389/fphar.2025.1498358

**Published:** 2025-03-26

**Authors:** Shan He, Yue Wu, Kaixian Lu, Heng Zhu, Xuan Wang, Yaoyao Qin, Huan Li, Lin Zeng, Jiaojiao Han, Xiangyang Zhou, Bin Zhang, Bo Tang

**Affiliations:** ^1^ College of Food and Bioengineering, Bengbu University, Bengbu, China; ^2^ School of Marine Sciences, Ningbo University, Ningbo, China; ^3^ Genepioneer Biotechnologies Co., Ltd., Nanjing, China

**Keywords:** sheep placenta extract, antioxidant, D-galactose, anti-aging, gut microbiota

## Abstract

**Introduction:**

Sheep placenta extract (SPE) is a representative traditional medicinal substance that exhibits multiple experimentally validated physiological properties, including anti-aging effects, wound healing acceleration, antioxidant activity, and anti-inflammatory mechanisms. However, the mechanism by which SPE influences the delay of aging is still not yet clear.

**Methods:**

Exploring the effects of sheep placenta extract on D-gal induced senescence in a mouse model of aging by macrogenomics and metabolomics.

**Results:**

In the serum of aging mice treated with SPE, the levels of antioxidant function such as superoxide dismutase (SOD), glutathione peroxidase (GSH-Px), and catalase (CAT) were notably higher compared to those in the blank group, whereas malondialdehyde (MDA) levels decreased. We revealed that SPE alleviated the changes in gut microbiota caused by aging in mice, with a significant decrease in the *Firmicutes/Bacteroidetes* (F/B) ratio in the gut. Furthermore, *Akkermansia muciniphila* (*A. muciniphila*), which is known for its regulating immune response and potential anti-aging effects, showed a significant increase of 1177.94%. The analysis of UHPLC-QE-MS combined with orthogonal partial least squares discriminant analysis (OPLS-DA) screening of differential metabolites in mouse serum metabolic profiles revealed a significant upregulation of *cis*-5,8,11,14,17-eicosapentaenoic acid (EPA) and triptolide in serum metabolites, following SPE treatment, which are commonly believed to have immunosuppressive, anti-inflammatory, anti-proliferative, and anti-tumor effects.

**Discussion:**

The role of SPE in ameliorating aging may be associated with the increased abundance of A. muciniphila in the gut microbiota and the accumulation of two metabolites, EPA and triptolide, in the serum.

## Graphical Abstract

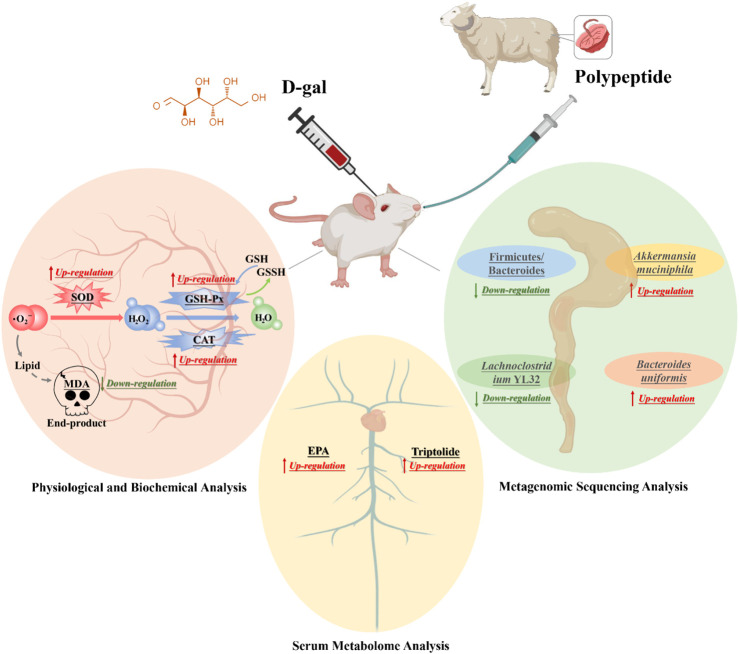

## 1 Introduction

The gut microbiome, comprising trillions of bacteria, archaea, eukaryotes, and viruses, has coevolved with the host in mutualistic symbiosis (Zhao et al., 2025). The diverse gut microbiota have a reciprocal and coevolutionary relationship with the host, helping in maintaining intestinal integrity, regulating host immunity, and providing direct and indirect pathogen protection (Du et al., 2022). The studies have shown that gut microbiota plays a crucial role in regulating health and lifespan by improving damaged intestinal barrier function (Nie et al., 2024; Zhao et al., 2022). Age-related alterations in the gut environment can precipitate chronic inflammation, metabolic dysfunction, and various diseases, thereby affecting the aging process (Kong et al., 2023).

The mechanisms of aging are complex and remain not fully understood. Previous studies have shown that the biological aging process is driven by multiple interrelated mechanisms, such as oxidative stress, inflammatory status, and autophagy function, which work together through various signaling pathways (Wu et al., 2021; Ling et al., 2022; Chen et al., 2022). The impact of gut microbiota on aging may be due to the fact that aging leads to a decline in the body’s immune system function and causes natural disturbances in microbial composition, known as dysbiosis of the gut microbiota (gut dysbiosis) (Golomb et al., 2020). Related literature research suggests that bioactive compounds from traditional Chinese medicine or natural products may be effective and proposes safe strategies for preventing aging and age-related diseases (Zhou et al., 2021; Shen et al., 2022; Lei et al., 2022; Kumar et al., 2022; Song and Zhang, 2023; Li et al., 2025; Li et al., 2024; Niu et al., 2024).

The placenta is an important organ in mammals that nurtures new life forms and plays a crucial role in ensuring the normal development of the fetus (Pogozhykh et al., 2018). The placenta contains various nutrients and bioactive components required for fetal growth and development, such as hormones, amino acids, nucleic acids, proteins, vitamin growth factors, and cytokines (Brett et al., 2014; Roy et al., 2022). Therefore, the placenta is an important medication in traditional medicine. For example, in Compendium of Materia Medica “Bencao Gangmu,” the human placenta is referred to as the “Ziheche,” which is believed to have the effects of nourishing blood and qi and nourishing liver and kidney (Shen et al., 2024; Silini et al., 2015). The prosperous development of animal husbandry has resulted in a substantial amount of placental waste. In recent years, there has been increasing research into the repurposing of this waste biomass, with a particular focus on the clinical therapeutic potential of active substances found in sheep placentas. Currently, research on the application of placental extracts includes promoting wound healing, liver disease, anti-inflammatory, antioxidant, anti-cancer, anti-viral, immune regulation, promoting hair growth, and delaying aging (Kim et al., 2022; Ghoneum and El-Gerbed, 2021; Han et al., 2013; Joshi et al., 2020; Lee et al., 2011). Placenta extracts have excellent research value in the field of animal anti-aging (Choi et al., 2014). However, the anti-aging effects of SPE, particularly its impact on the gut microbiota and oxidative stress of aging mice, have not been explored.

D-gal-treated mice are widely regarded as an ideal animal model for studying aging intervention drugs as they can induce physiological states similar to natural aging, such as decreased antioxidant enzyme activity and increased levels of free radicals (Xia et al., 2020). Measure the physiological and biochemical changes, histopathological changes, and gut microbiota of mouse serum and tissues, and explore their possible mechanisms of action through experiments such as metagenomics sequencing and UHPLC-QE-MS serum metabolomics.

## 2 Materials and methods

### 2.1 Materials and reagents

Fresh placenta of normal and healthy Hu sheep was collected from a Hu sheep breeding farm in Bengbu City, Anhui Province, China. The blood and dirt were immediately washed with physiological saline after removing the placenta and stored at −20°C. SPE was prepared by referring to the relevant literature and making slight modifications (Shen et al., 2022; Nensat et al., 2021; Park et al., 2015). First, the placenta was thawed and chopped at room temperature. Then, deionized water was added in a solid–liquid ratio of 1:2, and the placenta was homogenized using a high-speed homogenizer at 4°C (FSH-II, Jiangsu, China). Next, the homogenate solution was let to freeze and thawed three times at −20°C, centrifuged at 12,000 rpm for 20 min (Sigma 2-16 K, Sartorius, Germany), the supernatant was collected, filtered through ultrafiltration membrane, and the filtrate was freeze-dried to prepare SPE (Christ alpha 1-2 LD plus, Osterode am Harz, Germany). The SPE powder, procured through freeze-drying, was promptly sealed and preserved at −20°C for an extended duration. Anticipating its utilization, the material was thawed within a refrigerator set at a temperature of 4°C the day prior. The DPPH (1,1-diphenyl-2-picrylhydrazyl) radical scavenging ability of SPE was determined, and the results showed that the radical scavenging rate exceeded 40%.

D-gal (≥99%), Shanghai Aladdin Biochemical Technology Co. Ltd.; Tween 80 (purity ≥99%), Shanghai Aladdin Biochemical Technology Co. Ltd. (Shanghai, China). D-gal was dissolved in 0.9% saline and injected subcutaneously into the back of the neck of mice at a dose of 250 mg/kg body weight, and VE (50 mg/kg-d) was dissolved in distilled water containing 1% Tween 80 solutions. MDA, GSH-PX, CAT, and SOD kits, Nanjing Jiancheng Bioengineering Institute (Nanjing, China). All other chemicals and reagents used in this study were of analytically pure grade.

### 2.2 Animals and treatment

The aging model of Kunming mice treated with D-gal was studied using the method described in the reference literature (Wang D. et al., 2022; Zhang et al., 2023; Qian et al., 2021; Li et al., 2020). Forty male Kunming mice (SPF, body weight: 20 ± 5 g, age: 5–6 weeks) were purchased from Henan SKobes Biotechnology Co., LTD. (Henan, China; License number SCXK2020-0005), and the animal feed (no D-gal) was also purchased by this company. The animals that were housed in an animal house (an ambient temperature of 23°C ± 2°C, relative humidity of 55% ± 10%, and light/dark automatic lighting cycle of 12 h) had free access to standard diet and water. All experimental procedures and animal care were carried out in accordance with the Care and Use Guidelines, and the mice were allowed to acclimatize to the environment for 2 weeks prior to the experiment, and animal experiments were carried out in accordance with the Care and Use Guidelines for Laboratory Animals; the whole animal experiment was approved by the Ningbo University Laboratory Animal Center under permit number No. SYXK (ZHE 2008-0110). After 2 weeks of a normal dietary adaptation period, the mice were randomly divided into four groups (n = 9/group). The groups were as follows: normal control group (Control, CK), aging model group (Aging, A), positive control group (Vitamin E, V), and aging treatment group (Polypeptide, P). The CK group was established as a benchmark to precisely evaluate the actual impact of the intervention factors. Throughout the experiment, this group did not receive any experimental intervention but shared identical rearing conditions and living environments with the treatment groups. These mice were managed based on standardized procedures, receiving distilled water by gavage (0.5 mL/day) and subcutaneous saline injections (12.5 mL/kg-BW-d). The A group was developed using D-galactose (D-gal)-induced aging. Mice in this group were administered subcutaneous injections of D-gal (250 mg/kg-BW-d) and received distilled water via gavage (0.5 mL/day). The V group consisted of mice subjected to D-gal-induced aging and subsequently treated with vitamin E as the positive control. These mice received subcutaneous injections of equivalent amounts of D-Gal (250 mg/kg-BW-d) and were administered vitamin E dissolved in a 1% Tween 80 solution by gavage (50 mg/kg-BW-d). The P group aimed to investigate the potential of SPE in delaying aging and enhancing bodily functions. Mice in this group underwent D-gal-induced aging and were then treated with SPE. They received subcutaneous injections of D-Gal (250 mg/kg-BW-d) and were administered SPE dissolved in distilled water via gavage (0.5 mL/animal/day). All groups followed the experimental protocol for a duration of 6 weeks, guided by the results of *in vitro* free radical pretests.

### 2.3 Biochemical tests and histopathological analysis

Serum biochemical assay: mice were anesthetized with ether after weighing, and blood was collected from the eyeballs and immediately placed in sodium heparin-treated centrifuge tubes and centrifuged at 1040 ×g for 10 min at 4°C. After centrifugation, 200 μL of the blood supernatant was taken and placed into clean centrifuge tubes, and the rest of the supernatant was stored in another −80°C, and then, MDA, SOD, CAT, GSH-Px, and other indicators were detected, according to the manufacturer’s instructions and reference (Yu et al., 2022; Yu et al., 2021).

Morphological analysis of organ tissues: histopathological analysis of brain tissues was performed by H&E staining. The colon was isolated, immersed in 4% paraformaldehyde (pH 7.4) for 24 h, and then embedded in paraffin (5 μm thick). Organ histopathological changes were observed by scanning with a light microscope (NanoZoomer 2.0-RS, Hamamatsu, Japan)

### 2.4 Mouse serum metabolomics experiment

#### 2.4.1 Serum sample treatment

The serum sample (100 μL) was placed in the EP tubes and resuspended with pre-chilled 80% methanol and 0.1% formic acid by well vortex. Then, the samples were incubated on ice for 5 min and centrifuged at 15,000 g, 4°C for 20 min. Some of the supernatant was diluted to a final concentration containing 53% methanol by LC-MS grade water. The samples were subsequently transferred to a fresh Eppendorf tube and then were centrifuged at 15,000 g, 4°C for 20 min. Finally, the supernatant was injected into the LC-MS/MS system analysis.

#### 2.4.2 UHPLC-MS/MS analysis

Metabolomics is an analytical approach that employs advanced analytical instruments characterized by high separation efficiency, superior sensitivity, and minimal detection limits to identify a comprehensive range of metabolites within a specified sample. The UHPLC-MS/MS method has emerged as a pivotal analytical platform for the comprehensive analysis of metabolites in biological samples, owing to its exceptional reproducibility and sensitivity (Hu et al., 2021; Wu et al., 2018; Yang et al., 2024). In this study, UHPLC-MS/MS analyses were performed using a Vanquish UHPLC system (Thermo Fisher Scientific, Germany) coupled with an Orbitrap Q Exactive TM HF-X mass spectrometer (Thermo Fisher Scientific, Germany) in Gene Denovo Co., Ltd. (Guangzhou, China). Samples were injected into a Hypesil GOLD column (100 × 2.1 mm, 1.9 μm) using a 17-min linear gradient at a flow rate of 0.2 mL/min. The eluents for the positive polarity mode were eluent A (0.1% FA in water) and eluent B (methanol). The eluents for the negative polarity mode were eluent A (5 mM ammonium acetate, pH 9.0) and eluent B (methanol). The solvent gradient was set as follows: 2% B, 1.5 min; 2%–100% B, 12.0 min; 100% B, 14.0 min; 100%–2% B, 14.1 min; 2% B, 17 min. The Q Exactive TM HF-X mass spectrometer was operated in the positive/negative polarity mode with a spray voltage of 3.2 kV, capillary temperature of 320°C, sheath gas flow rate of 40 arb, and aux gas flow rate of 10 arb.

#### 2.4.3 Data processing and metabolite identification

The raw data files generated by UHPLC-MS/MS were processed using Compound Discoverer 3.1 (CD3.1, Thermo Fisher) to perform peak alignment, peak picking, and quantitation for each metabolite. The main parameters were set as follows: retention time tolerance, 0.2 min; actual mass tolerance, 5 ppm; signal intensity tolerance, 30%; signal/noise ratio 3; and minimum intensity 100,000. After that, peak intensities were normalized to the total spectral intensity. The normalized data were used to predict the molecular formula based on additive ions, molecular ion peaks, and fragment ions, and then, peaks were matched with the mzCloud (https://www.mzcloud.org/), mzVault, and Mass List databases to obtain the accurate qualitative and relative quantitative results.

### 2.5 Data processing

Differential metabolites were screened using Student's t-test, variable importance in the projection (VIP), and fold change (FC) of the first principal component of the OPLS-DA model, with the screening criteria (with replicates) as between-groups P-value (P-value) less than 0.05, VIP greater than 1, FC as multiplicity of differences, greater than 1 upward, and less than 1 downward. The screening criteria (with replicates) were P-values less than 0.05, VIP greater than 1, and FC as the multiplicity of difference, greater than 1 for upregulation and less than 1 for downregulation.

## 3 Results

### 3.1 Changes in daily behavior

During the 6-week experimental period, the daily appearance and behavioral activities of the mice were observed and recorded weekly, and the results showed that no pathological changes, such as vomiting and refusal to eat, were observed in all groups. The mice in the CK group were in a good mental state, lively, active, and agile and had a healthy diet. In addition, 6 weeks after the injection of D-gal, the mice in the A group appeared to clearly exhibit negative symptoms, including dull fur color and easy to fall off, showed slow movement, gradual decrease in food intake, lagging response, and depressed spirit, which indicated that the senescent mouse model was successfully established by subcutaneous injection. Compared with the model group, the aging characteristics of V group and P group mice were reduced to different degrees, and the daily appearance and behavioral activities of V and P groups were similar, with the fur being slightly dull and the response slightly sluggish, but the aging characteristics were significantly reduced (*P* < 0.05) in comparison with the A group mice.

### 3.2 Changes in body weight and organ index

**FIGURE 1:**
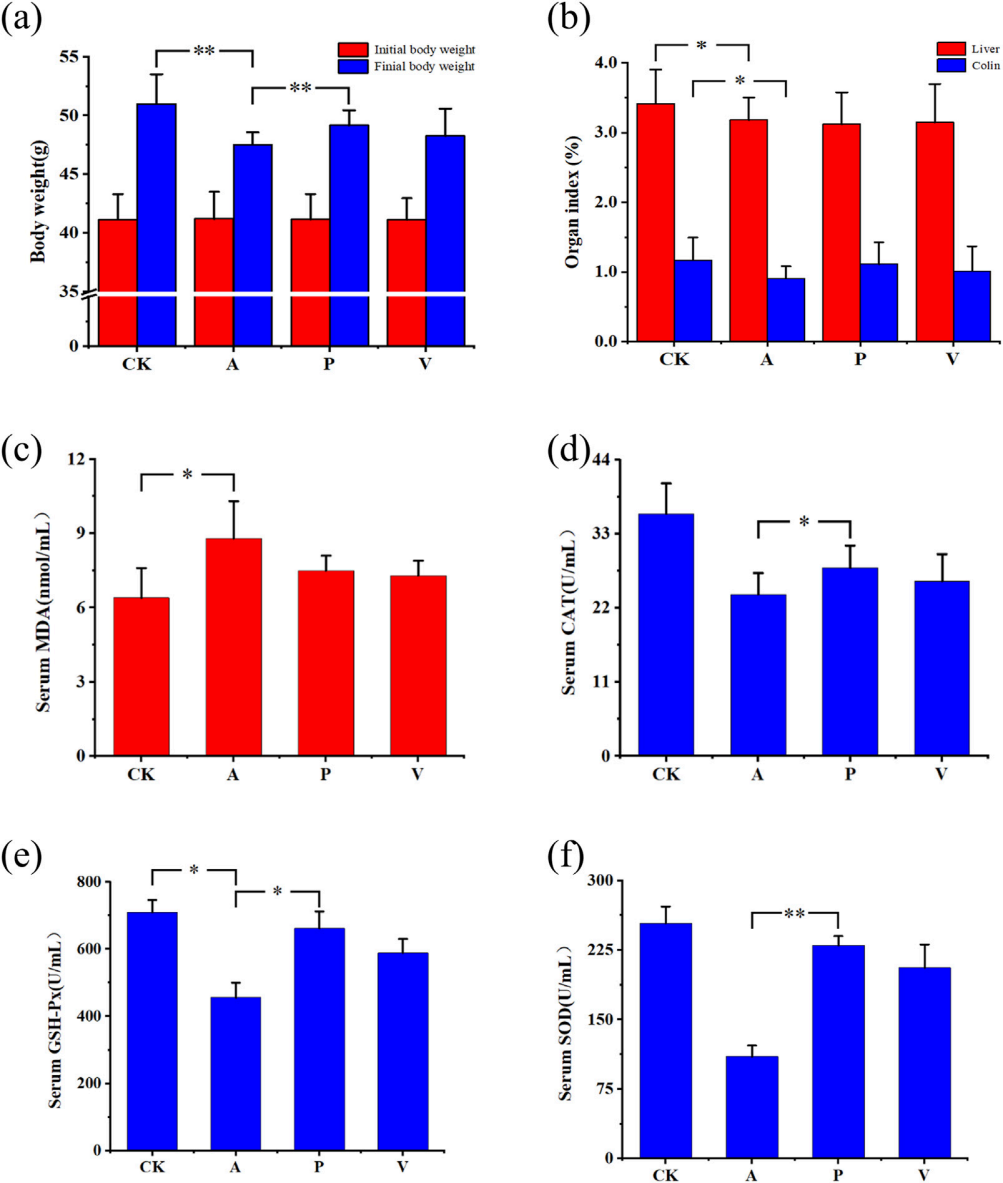
Changes in body weight, organ indices, and serum biochemical indices in mice. **(A)** Changes in body weight; **(B)** changes in organ indices; **(C)** change in serum MDA indices; **(D)** changes in serum CAT indices; **(E)** changes in serum GSH-Px indices; and **(F)** changes in serum SOD indices; significant differences among groups, *P < 0.05, **P < 0.01.

At the initial stage of D-gal injection, groups A, P, and V all showed weight loss, followed by gradual recovery and stable growth of body weight, but the overall level did not reach the level of CK group. As can be seen from Figure 1A, after 6 weeks of experimental cycle, D-gal injection affected the body weight growth of mice in groups A, P, and V. The antioxidant peptide was able to alleviate the effect of D-gal injection, which gradually restored the body weight of mice in group P to the normal level. The body weights of mice in group A were significantly lower than those of mice in group CK (*P* < 0.05), and at the same time, the body weights of mice in group P were significantly lower than those of mice in group A (*P* < 0.05).

After the mice were dissected, organs such as the brain, liver, and colon were taken and washed with saline, and filter paper was weighed after absorbing the surface moisture to calculate the organ coefficients, which were calculated by the following formula: organ coefficient (%) = organ weight (mg)/mouse weight (g)×100%, and the changes in organ in-dices are shown in Figure 1B. After 6 weeks of injection of D-Gal, liver organ coefficients of group A decreased by 6.74% compared to that of group CK, and those of group P decreased by 8.5% compared to that of group CK. Coefficients decreased by 8.50% compared to the CK group, and liver organ coefficients decreased by 7.79% compared to the CK group in group V. Colon organ coefficients decreased by 22.42% compared to the CK group in group A, colon organ coefficients decreased by 5.18% compared to the CK group in group P, and colon organ coefficients decreased by 13.61% compared to the CK group in group V. The differences in liver and colon organ coefficients in group A and CK groups were both significant. It is evident that SPE influences the growth and development of liver and colon organs, and the effect of aging on the growth and development of the colon in group P was substantially reduced.

### 3.3 Changes of biochemical indexes in mice

The oxidative stress indexes of three antioxidant enzymes SOD (superoxide dismutase), CAT (catalase), and GSH-Px (glutathione peroxidase) related to serum and natural defense system against oxidative damage, as well as the MDA content was analyzed to determine the antioxidant effects of antioxidant peptides on D-gal-induced senescent mice. As can be seen from Figures 1C–F, SOD (*P* < 0.05), GSH-Px, and CAT (*P* < 0.05) activities were significantly reduced, and MDA content was significantly elevated (*P* < 0.05) in group A compared with group CK. Group P was able to significantly increase the SOD activity (P < 0.05), GSH-Px activity (*P* < 0.05), and CAT activity , but the MDA content was significantly reduced. In comparison to group A, group P demonstrates an enhancement in the expression levels of SOD, CAT, and GSH-Px while concurrently decreasing the expression level of MDA, notably with the most significant impact observed on SOD expression (Supplementary Figure S1). Relative to the CK group, the SOD expression level within the P group closely resembles that of the CK group, achieving 91% of the CK group’s level, and the GSH-Px expression reaches 93% of the CK group’s level. The results indicate that the antioxidant peptide of lake sheep placenta significantly increased the activities of SOD, GSH-Px, and CAT and decreased the content of MDA, effectively preventing oxidative stress-induced oxidative damage.

### 3.4 Histopathological changes in the mouse colon

**FIGURE 2:**
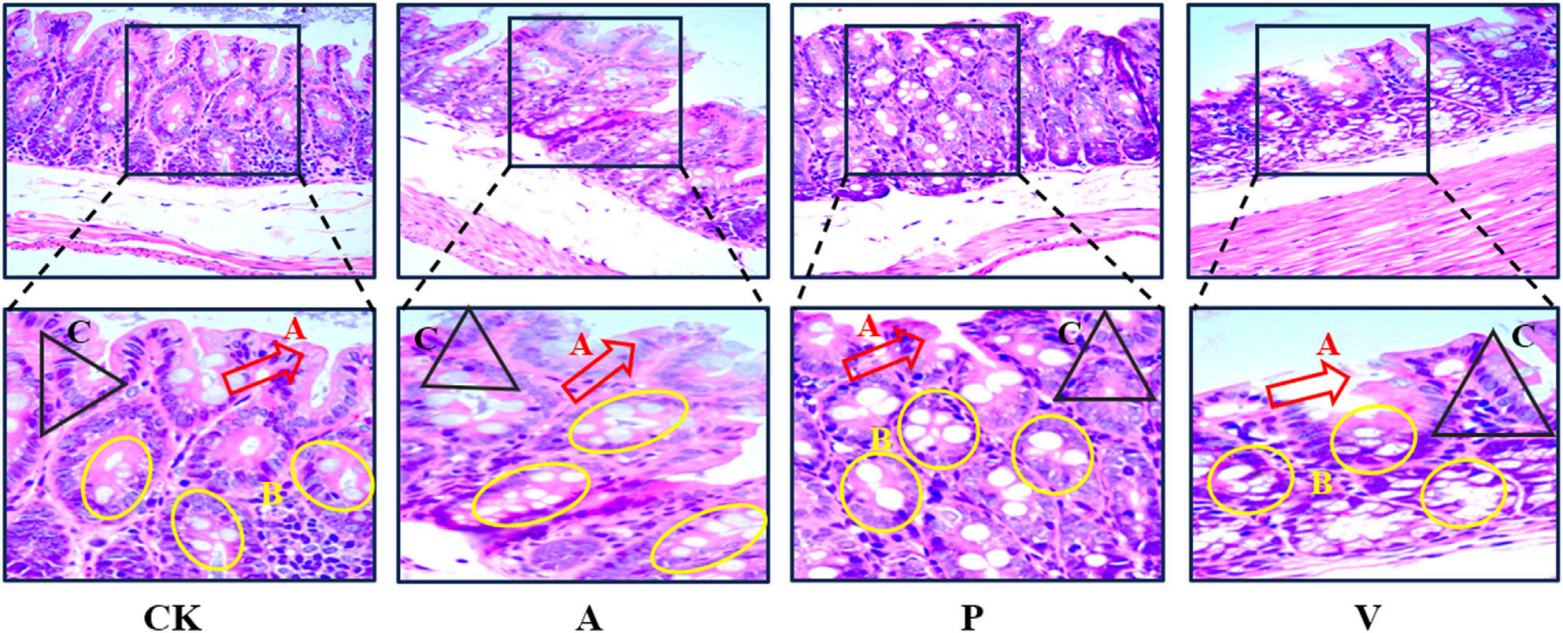
H&E staining of colon tissue (400×); A, epithelial layer; B, cup-shaped cells; C, cells with regular arrangement.

The images of H&E stained mouse colon tissue sections were used to analyze the histopathological changes of colon tissue under different reagent treatments, and the staining of the normal control group (CK group) showed the integrity of the epithelial layer, the number of goblet cells (goblet cells) was normal and uniformly distributed, and the cells were arranged in a regular and neat manner. The epithelial layer of the senescence model group (group A) showed some continuity disruption damage, uneven distribution of goblet cells, depletion, and disorganized and irregular cell arrangement. In the senescence treatment group (group P), pathological damage to the colon was significantly attenuated, and the integrity of the epithelial layer was improved, the number of cup cells was increased, and the cell arrangement was more regular than that of group A, but the overall situation was not as good as that of group CK, as shown in Figure 2.

### 3.5 Changes in mouse gut microorganisms

**FIGURE 3:**
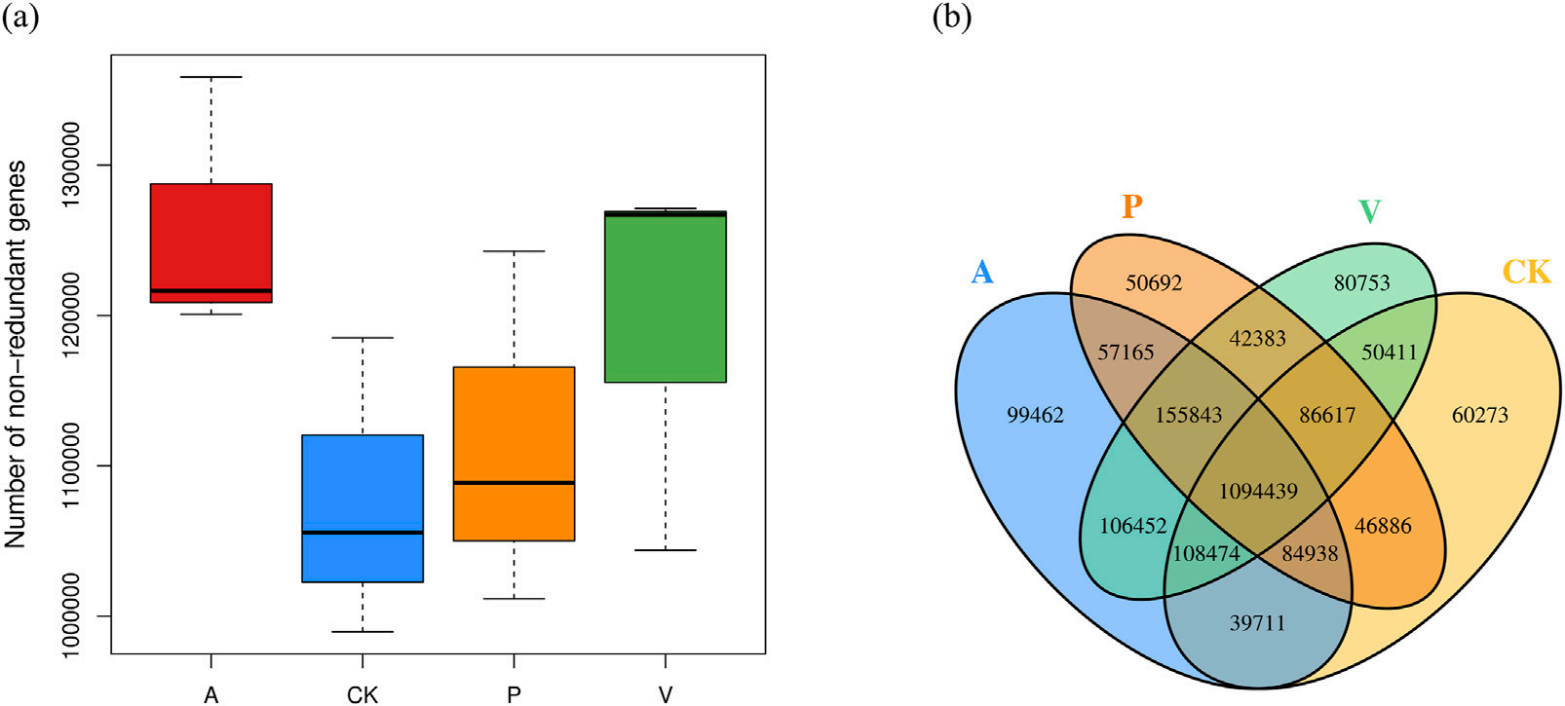
**(A)** Box plot of differences in the number of genes between groups and **(B)** Venn’s plot of the number of shared genes among groups.

In order to examine the differences in the number of genes between groups, the box plot of the differences in the number of genes between groups was drawn, and the results are shown in Figure 3A; In order to examine the distribution of the number of genes between the specified groups and to analyze the common and specific information on the genes between the different groups, Venn Graph was drawn, and the results are shown in Figure 3B. It is obvious that there are significant differences in the genes between groups, as can be seen from Figure 3. Alpha diversity and beta diversity are widely recognized metrics used to quantitatively summarize species diversity (Webster et al., 2022; Zhu et al., 2024; Parker et al., 2022). We computed the average values of alpha diversity and beta diversity for the microbial community composition across each sample group (Supplementary Figures S2, S3). The alpha diversity analysis revealed that with an increase in the sequencing depth, the curve exhibited a tendency to flatten, suggesting that the sequencing data obtained were adequate. The beta diversity analysis results indicated that group A displayed significant differences compared to other groups, suggesting that the composition and variety of bacterial communities in its samples were more extensive than those in other groups. Additionally, the P and V groups demonstrated a certain level of similarity, with overlapping samples within their respective groups.

**FIGURE 4:**
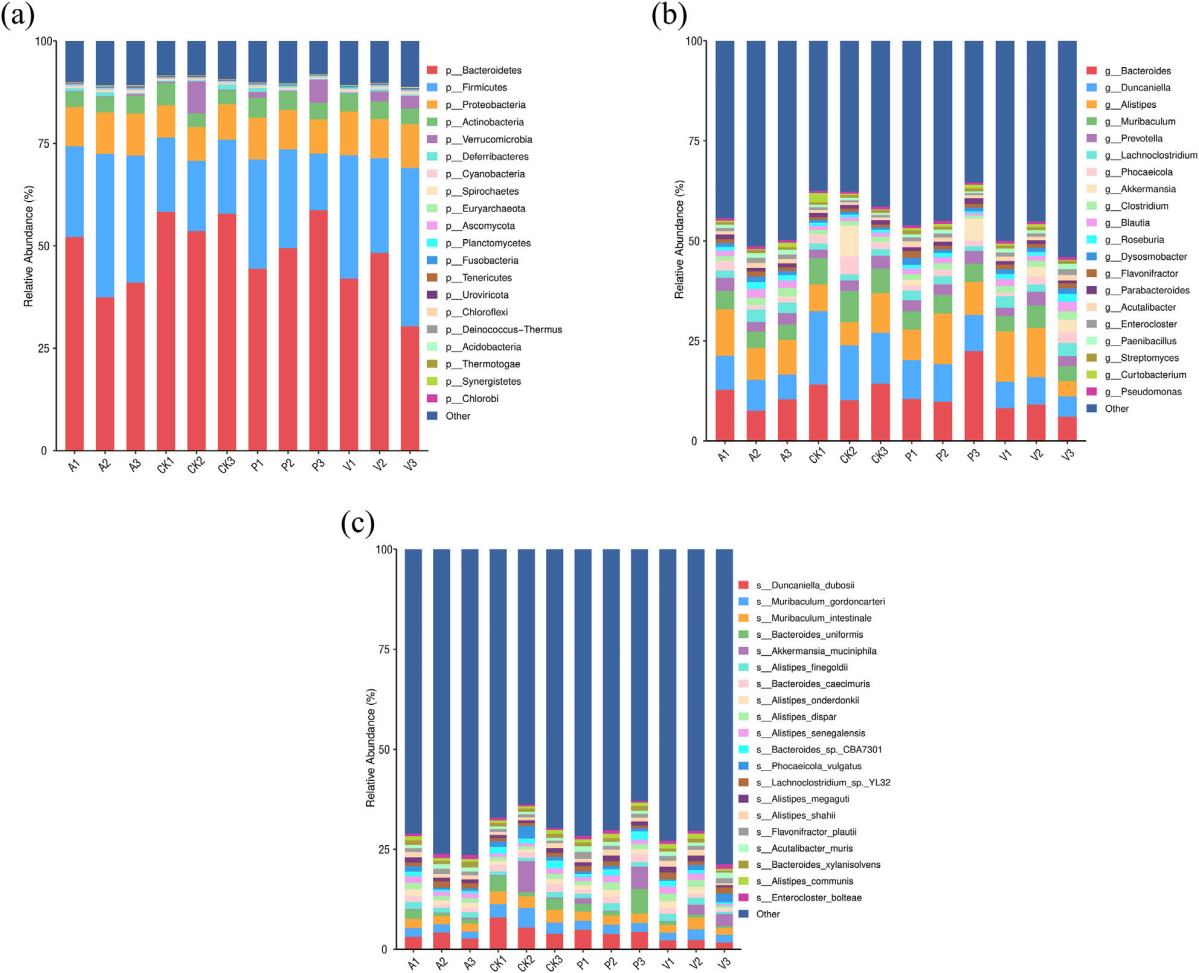
Changes in the intestinal microflora of mice. **(A)** Comparison of dominant flora at the phylum level in four groups of mice; **(B)** comparison of dominant flora at the genus level in four groups of mice; and **(C)** comparison of dominant flora at the species level in four groups of mice.

By comparing the differences in intestinal flora components among the four groups of mice in CK, A, P, and V groups, it was found that the four groups of mice were close to each other in terms of the types of major phyla at the level of phylum categorization, in which the total proportion of the five phyla of Bacteroidetes, Firmicutes, Proteobacteria, Actinobacteria, and Verrucomicrobia were all more than 85%, and Bacteroidetes and Firmicutes were both the major phyla with a proportion of more than 70%. For the abundance of the phylum Bacteroidetes, it was 56.56% in group CK, 43.51% in group A, 50.82% in group P, and 40.19% in group V. For the abundance of the Firmicutes, it was 17.77% in group CK, 29.34% in group A, 21.54% in group P, and 30.66% in group V. For details, as shown in Figure 4. For the ratio of Firmicutes/Bacteroidetes (F/B), it was 31.41% in group CK, 31.41% in group 67.56% in group A, 42.39% in group P, and 76.30% in group V. This shows that the antioxidant peptide adjusted the flora structure of senescent mice to a certain extent by reducing the F/B value.

At the genus level, the dominant intestinal flora in the four groups of mice were *Bacteroides*, *Duncaniella*, *Alistipes*, *Muribaculum*, *Prevotella*, *Lachnoclostridium*, *Phocaeicola*, *Akkermansia*, *Clostridium*, *Blautia*, and *Roseburia* spp. Among these dominant bacteria, in group P compared to group A, the percentage of Bacteroides, *Duncaniella*, *Muribaculum*, *Akkermansia*, *Curtobacterium*, and *Duchenne*’s percentage even exceeded that of the CK group. In addition, *Lachnoclostridium*, *Blautia*, and *Rossella spp*. accounted for a significant decrease in content in group P compared to group A, with a decrease of 25.31%, 35.19%, and 37.10%, respectively. The percentage is close to the CK group, the change of the percentage of these bacteria is bound to affect the intestinal structure and function; at the same time, the percentage of elevated 1164.07% *Akkermansia spp*. is likely to have a special effect.

At the species level, Duncaniella_dubosii, Muribaculum_gordoncarteri, Muribaculum_intestinale, *Bacteroides*_uniformis, Akkermansia_muciniphila, Alistipes_finegoldii, Bacteroides_caecimuris, Alistipes_onderdonkii, Alistipes_dispar, Alistipes_senegalensis, Bacteroides_sp._CBA7301, Phocaeicola_vulgatus, Lachnoclostridium_sp._YL32, Alistipes_megaguti, Alistipes_shahii, Flavonifractor_plautii, Acutalibacter_muris, Bacteroides_xylanisolvens, Alistipes_communis, and Enterocloster_bolteae were the abundance-dominant species. Duncaniella_dubosii was the most dominant species in all the groups, with 5.76%, 5.36%, 4.36%, and 4.37% in groups CK, A, P, and V, respectively, 3.36%, 4.37%, and 2.10%. The relative abundance share of Duncaniella_dubosii species in group P was elevated by 30.11% compared to group A (Liu et al., 2023). The two Muribaculum genera, Muribaculum_gordoncarteri, and Muribaculum_intestinale were the second and third most abundant strains in the intestines of the four groups of mice, and overall, the percentage of both strains in group P was significantly higher than that in group A. Notably, *Akkermansia muciniphila*, which has a positive effect function on regulating the intestinal barrier, increased its relative abundance percentage by 1,177.94% in group P compared to group A. *Bacteroides*_sp._CBA7301 strain increased its relative abundance percentage by 99.44% in group P compared to group A. The relative abundance percentage of *Bacteroides*_caecimuris in group P was enhanced by 47.63% compared to group A. The percentage of *Bacteroides*_uniformis strains in group P was enhanced by 123.11% compared to group A. In contrast to the trend of these percentages, the relative abundance percentage of *Lachnoclostridium* sp. YL32 strains in group P was enhanced by 123.11% compared to group A. In contrast to the trend of these percentages, *Lachnoclostridium* sp. YL32 strains in group P. The relative abundance of *Lachnoclostridium* sp. YL32 decreased by 19.26% compared to group A. Among the top 20 dominant strains in the four groups of mice, Alistipes had the highest number of species, with seven species, and the percentage of these seven species was relatively stable in group P compared with group A, with five species’ percentage changes of 10% or less, which were small, 10.43% and 16.17%. Collectively, these findings demonstrate that SPE has a positive improvement effect on age-related gut microbiota dysbiosis.

### 3.6 Mouse serum metabolomics results

**FIGURE 5:**
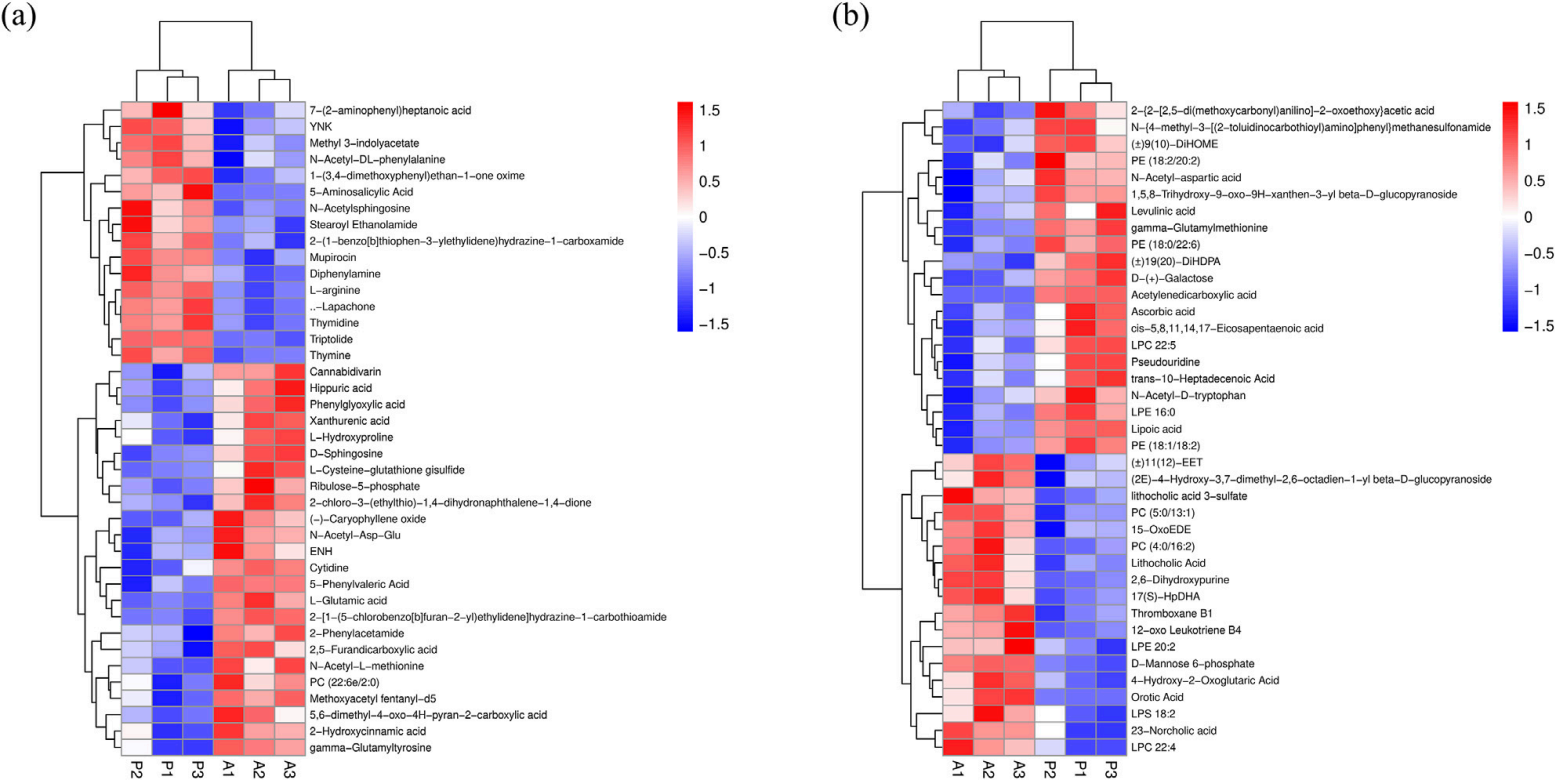
Hierarchical clustering analysis. **(A)** Positive ion mode hierarchical clustering analysis. **(B)** Negative ion mode hierarchical clustering analysis.

Serum metabolic profiles were examined using UHPLC-QE-MS in positive and negative ion modes. Potential biomarkers in serum were searched for using OPLS-DA to differentiate between senescent and control groups, as well as P and senescent groups. Model data were randomized multiple times (n = 200) to validate the model. The results showed that the model does not produce overfitting and is reliable. Using the importance of the variable in the projection (VIP) > 1 and *P* < 0.05 as the screening condition for differential metabolites, 63 differential variables related to aging were identified in group A compared to group CK, and 79 differential variables related to aging were identified in group P com-pared to group A by positive and negative ion pattern analyses (Supplementary Table S1), of which *cis*-5,8,11,14,17- eicosapentaenoic acid, triptolide, 7-(2-aminophenyl)heptanoic acid, methoxyacetyl fentanyl-d5, (−)-caryophyllene oxide, and LPC 22:4, the six metabolites, showed specificity (Figure 5; Supplementary Table S1), three metabolites, *cis*-5,8,11,14,17-eicosapentaenoic acid, triptolide, and 7-(2-aminophenyl)heptanoic acid, were significantly downregulated in the A group compared to the CK group, and significantly upregulated in the P group compared to the A group. The changes of three metabolites, methoxyacetyl fentanyl-d5, (−)-caryophyllene oxide, and LPC 22:4, were the opposite, significantly upregulated in group A compared to group CK and significantly downregulated in group P compared to group A. The changes of three metabolites, methoxyacetyl fentanyl-d5, (−)-caryophyllene oxide, and LPC 22:4, were significantly downregulated in group P compared to group A. Moreover, among the significantly changed metabolites, triptolide showed a fold change of 0.21 in group A compared to group CK and a fold change of 3.09 in group P compared to group A. The fold change of *cis*-5,8,11,14,17-eicosapentaenoic acid was similarly significant, with a fold change in group A compared to group CK. Similarly, the FC of *cis*-5,8,11,14,17-eicosapentaenoic acid reached 0.64 in group A and 1.34 in group P compared to group CK, indicating that these two metabolites are likely to be the metabolic accumulation of some important functions and responses.

## 4 Discussion

Antioxidant peptides extracted from natural products have significant free radical scavenging effects and are increasingly being used in the food and health product industries (Li et al., 2025; Olivares-Galván et al., 2020; Yu and Li, 2025). Although the exact anti-aging mechanism of this active extract remains uncertain, our experimental research indicates that antioxidant peptides derived from sheep placenta can significantly enhance the activity status and organ index of D-galactose-induced aging mice, effectively retarding the aging process. Specifically, these antioxidant peptides exhibit a highly efficient capacity to neutralize free radicals, which may optimize the gut microbiota structure in aging organisms. This helps alleviate the oxidative stress response triggered by D-galactose intake in aging mice, ultimately contributing to an overall improvement in aging conditions (Wang J. et al., 2022; Li et al., 2023; Matsuoka et al., 2024).

Reactive oxygen species (ROS) are essential for the normal functioning of biological organisms, but they can also cause oxidative and lipid peroxidation damage throughout the aging process (Anik et al., 2022; Shi et al., 2022). The widely accepted free radical theory of aging (FRTA) posits that the cumulative oxidative damage caused by ROS is a primary contributor to the aging process. Antioxidants can effectively eliminate free radicals and play a crucial role in preventing and treating many age-related diseases (Shields et al., 2021; Santos et al., 2023). Metabolomics based on UHPLC-MS/MS results indicate that the serum of mice treated with SPE contains various substances with antioxidant functions, which play a crucial role in alleviating the oxidative stress response of the body. Significant changes in *cis*-5,8,11,14,17-eicosapentaenoic acid and triptolide were found in the results of serum metabolism in mice. EPA plays a wide range of roles in the treatment of autoimmune deficiencies and inflammation induced by autoimmune deficiencies, and a large number of studies have also demonstrated its effects on the treatment of lung disease, renal disease, type 2 diabetes mellitus, colorectal ulcers, and segmental ileitis (Xie et al., 2015). Another study suggests that EPA plays a beneficial role in maintaining energy metabolism and lipid homeostasis. Polyunsaturated fatty acids like EPA are primarily incorporated into the diet to promote energy metabolism, which can help delay the aging process (Xiong et al., 2024).

The NF-κB transcription factor is a central regulatory factor for immunity and inflammation, playing an important role in coordinating immune and inflammatory responses (Capece et al., 2022). The NF-κB pathway is currently widely used to regulate cellular inflammatory responses (Li et al., 2019). Triptolide is a tricyclic diterpenoid oxide and suppresses the inflammatory response of cells via inhibition of NF-κB activation (Song et al., 2019). Compared with group A, group P showed a 3.09-fold enhancement of triptolide FC and a 1.34-fold enhancement of EPA, which are very helpful in enhancing anti-inflammatory effects enhancement (Mak et al., 2009). The results of the present study demonstrate that dietary supplementation with antioxidant peptides significantly enhances anti-inflammatory capacity in senescent mice, concomitant with a marked alleviation of colonic inflammation. These metabolites influence or even directly participate in the generation of inflammation and the aging process.

The association between aging and gut microbiota dysbiosis is characterized by elevated expression of harmful bacteria and diminished expression of beneficial bacteria in the damaged gut. Moreover, bacterial products can infiltrate the host circulation via the compromised gut barrier, further intensifying this imbalance (Liu et al., 2020). At the level of the intestinal flora phylum, an elevated or decreased ratio of F thick-walled phylum/B mimic phylum is often regarded as an ecological disorder because an elevated *F/B* ratio is often closely associated with obesity, metabolic disorders, and a decreased *F/B* ratio is often associated with inflammatory bowel disease associated with immune-inflammatory disorders, depression, and Alzheimer’s disease (Cao et al., 2021). Relevant literature shows that there are significant differences in the composition of the gut microbiota at the level of major microbial phyla among different age groups, and the F/B ratio tends to increase with age (Vaiserman et al., 2020). In this study, antioxidant peptides could regulate the abundance of the intestinal flora of *Bacteroides* thick-walled and *Bacteroides* anomalies and reduce the *F/B* ratio in group P. Compared with the F/B ratio in group A (67.56%), the *F/B* ratio of group P decreased to 42.39%, which was close to that of group CK (31.41%), which showed that antioxidant peptides were effective in improving the imbalance of the intestinal microbiota induced by the intake of D-gal. This result indicates that SPE’s anti-aging work can achieve its goal by affecting gut microbiota.

*A. muciniphila* is a bacterium that colonizes the intestinal mucosa of humans and rodents. Research has shown that higher levels of *A. muciniphila* are associated with a lower incidence of conditions such as intestinal inflammation, obesity, and diabetes. Consequently, in recent years, this bacterium has been investigated as an immunomodulatory probiotic for the treatment of autoimmune and chronic inflammatory diseases (Rodrigues et al., 2022). At the genus level, it was found that the abundance of several genera with specific functions showed significant differences. The abundance of *Akkermansia* spp. was elevated by 1164.07% in group P compared to group A. Combined with the stained pictures of the colon, we inferred that *Akkermansia* spp. is likely to play a key role in promoting the integrity of the intestinal barrier, regulating immune response, inhibiting inflammation, and other biological functions. Meanwhile, we observed a significant downregulation of the abundance percentage of Trichoderma spp. in group P com-pared with group A. *Lachnoclostridium* has a high abundance and prevalence among human gut microorganisms, and its abundance may be related to nutritional metabolism and gut health. The relative abundance of *Lachnoclostridium* spp. varies in different disease states, e.g., *Lachnoclostridium* spp. are higher in the gut flora of patients with ulcerative colitis and irritable bowel syndrome (Wang et al., 2018; Zhu et al., 2022; Ding et al., 2022). This implies that the abundance of *Lachnoclostridium* spp. can be used as a correlation of colonic injury. The significant decrease in the percentage of abundance of *Lachnoclostridium* spp. in group P compared to group A also proved that colonic inflammation was effectively alleviated in group P mice. At the species level, we observed that *Duncaniella dubosii* was the dominant species with the highest abundance, with 5.76%, 3.36%, 4.37%, and 2.10% in the CK, A, P, and V groups, respectively, and the relative abundance share of the P group was elevated by 30.11% compared to that of the A group. However, the potential function of *Duncaniella dubosii* is relatively unclear (Miyake et al., 2020); thus, more research is needed to decipher its complex multimodal disease mechanisms, as well as targeted studies of sub-type phenotypes. At the species level, the most noteworthy is *A. muciniphila*, which has the ability to improve systemic anti-aging metabolites and attenuate immune activation in accelerated aging mice (van der Ark et al., 2018; Li et al., 2016; Daniel et al., 2023), the relative abundance share of this strain in group P was elevated by 1177.94% compared to group A, which can also correspond to the result of the substantial increase in the abundance share at the genus level. Therefore, the remarkable effect of *A. muciniphila* in regulating intestinal homeostasis and alleviating colitis in mice plays a key role in the overall anti-aging effect in mice.

## 5 Conclusion

In summary, the results of the present study demonstrated that SPE can effectively regulate intestinal microbiota imbalance and metabolic disorders, improve aging status, and protect colon morphology and structure. Unlike single-function antioxidant drugs, SPE exhibits synergistic effects of antioxidant activity and regulation of gut microbiota through multiple mechanisms. The results of this study increase our understanding of the anti-aging mechanism of SPE and provide possibilities for the resource utilization of waste biomass.

## Data Availability

The original contributions presented in the study are publicly available. This data can be found at the NCBI (SRA) repository, accession number PRJNA1232994 (available at https://www.ncbi.nlm.nih.gov/sra/PRJNA1232994); and the MetaboLights repository, accession number MTBLS12312.
