# Supplementary material for: Effect of sheep placenta extract on D-galactose-induced aging mouse
Source: Front Pharmacol. 2025 Mar 26;16:1498358. doi: 10.3389/fphar.2025.1498358 (PMC11979192; doi:10.3389/fphar.2025.1498358)
Supplement: Supplementary file 1 [file DataSheet1.docx]

Supplementary Material

Effect of sheep placenta extract on D-galactose induced aging mouse

Shan He^1^, Yue Wu^1^, Kaixian Lu^1^, Heng Zhu^1^, Xuan Wang^1^, Yaoyao Qin^1^, Huan Li^1^, Lin Zeng^1^, Jiaojiao Han^2^, Xiangyang Zhou^3^, Bin Zhang^1^,* Bo Tang^1^,*

^1^ College of Food and Bioengineering, Bengbu University, Bengbu 233000, China

^2^ School of Marine Sciences, Ningbo University, Ningbo 315211, China

^3^ Genepioneer Biotechnologies Co., Ltd., Nanjing, 210000, China

*** Correspondence:**Correspondence: zhangbin207@163.com (B.Z.); tb@bbc.edu.cn (B.T.)

# Supplementary Tables

Table S1. Differential metabolites in group A compared to group CK

| Ion pattern | Peak | rt | VIP | Pvalue | FoldChange | significant |
| --- | --- | --- | --- | --- | --- | --- |
| POS | 2-(Formylamino)Benzoic Acid | 1.324 | 1.53567 | 0.009580234 | 0.738634206 | Down |
| POS | SM (d23:1/18:1) | 10.24 | 1.58838 | 0.0037864 | 0.70743134 | Down |
| POS | gamma-Glutamyltyrosine | 5.004 | 1.45638 | 0.035463049 | 0.794787824 | Down |
| POS | Isorhapontigenin | 5.735 | 1.39921 | 0.049036527 | 0.670475888 | Down |
| POS | SM (d29:0/15:0) | 10.246 | 1.49742 | 0.031809025 | 0.730484706 | Down |
| POS | 7-(2-aminophenyl)heptanoic acid | 6.611 | 1.48679 | 0.041259817 | 0.355969613 | Down |
| POS | 1-(4-benzylpiperazino)-2-(pyridin-2-ylamino)propan-1-one | 5.292 | 1.38461 | 0.046581016 | 0.482505819 | Down |
| POS | 2-(tert-butyl)-6,7-dimethoxy-4H-3,1-benzoxazin-4-one | 4.993 | 1.51791 | 0.009514616 | 0.51267725 | Down |
| POS | Kinetin | 5.023 | 1.4755 | 0.041294008 | 0.773595288 | Down |
| POS | p-Hydroxybenzaldehyde | 10.348 | 1.54427 | 0.009802338 | 0.711310275 | Down |
| POS | PC (18:2e/24:4) | 9.391 | 1.52969 | 0.048046382 | 0.566188124 | Down |
| POS | L-(-)-alpha-Amino-epsilon-Caprolactam | 9.641 | 1.40898 | 0.029311874 | 0.848771932 | Down |
| POS | Triptolide | 9.419 | 1.54019 | 0.041726744 | 0.209942345 | Down |
| POS | 4-(9H-fluoren-9-yl)pyridine | 4.906 | 1.54852 | 0.015056853 | 0.562027946 | Down |
| POS | Methamphetamine | 5.51 | 1.46591 | 0.025274661 | 0.815557798 | Down |
| POS | Stearoyl Ethanolamide | 6.84 | 1.49442 | 0.028000605 | 1.483146376 | Up |
| POS | 6,7,8-trimethoxy-2-(2-phenoxy-3-pyridyl)-4H-3,1-benzoxazin-4-one | 9.655 | 1.4217 | 0.021919638 | 1.392801818 | Up |
| POS | ACar 20:1 | 8.637 | 1.47004 | 0.032928285 | 1.262845057 | Up |
| POS | Biliverdin | 6.183 | 1.42465 | 0.024016304 | 1.538535113 | Up |
| POS | 3-Ureidopropionic acid | 1.476 | 1.59744 | 0.007504382 | 1.328282777 | Up |
| POS | ACar 10:1 | 5.991 | 1.43454 | 0.049129857 | 1.336894906 | Up |
| POS | ACar 10:2 | 5.832 | 1.3423 | 0.044543534 | 1.404212781 | Up |
| POS | N-Methylhydantoin | 1.458 | 1.45853 | 0.038495965 | 1.330894272 | Up |
| POS | ACar 20:2 | 8.207 | 1.49989 | 0.027916037 | 1.353698806 | Up |
| POS | ACar 22:6 | 7.512 | 1.54651 | 0.031838678 | 1.485909502 | Up |
| POS | Palmitoylcarnitine | 7.811 | 1.38385 | 0.043458026 | 1.246595835 | Up |
| POS | Methoxyacetyl fentanyl-d5 | 6.076 | 1.61221 | 0.003278337 | 1.512272971 | Up |
| POS | N-Isovalerylglycine | 5.368 | 1.44483 | 0.03197116 | 1.327604405 | Up |
| POS | Proline | 1.428 | 1.4501 | 0.037722326 | 1.100419837 | Up |
| POS | D-Glucose 6-phosphate | 1.351 | 1.56577 | 0.008424718 | 1.464684721 | Up |
| POS | N6,N6,N6-Trimethyl-L-lysine | 1.284 | 1.58736 | 0.022172071 | 1.30936068 | Up |
| POS | Linoleoyl ethanolamide | 9.36 | 1.47548 | 0.024238757 | 1.454061426 | Up |
| POS | DL-Î±-Aminocaprylic acid | 5.584 | 1.47615 | 0.049820154 | 1.207163278 | Up |
| POS | (-)-Caryophyllene oxide | 1.354 | 1.42677 | 0.041884145 | 1.248405607 | Up |
| POS | Thymidine | 4.91 | 1.45492 | 0.036008011 | 1.150245456 | Up |
| POS | DL-Arginine | 1.304 | 1.55093 | 0.032417154 | 1.280202954 | Up |
| POS | Guanidineacetic acid | 1.36 | 1.47176 | 0.00828196 | 1.31914463 | Up |
| POS | ACar 17:1 | 7.66 | 1.48998 | 0.021761121 | 1.348325485 | Up |
| POS | 3-(2-Naphthyl)-D-Alanine | 6.312 | 1.40145 | 0.039980499 | 1.593097896 | Up |
| POS | PC (18:5e/4:0) | 9.12 | 1.39341 | 0.036716482 | 1.395213663 | Up |
| POS | Tetrahydrocortisone | 6.096 | 1.61509 | 2.79783E-05 | 1.324402707 | Up |
| POS | Nicotinamide | 2.056 | 1.45604 | 0.032324849 | 1.226828851 | Up |
| POS | PC (20:2/20:3) | 10.639 | 1.53081 | 0.020607289 | 16.12653669 | Up |
| POS | PC (18:3e/2:0) | 9.276 | 1.49159 | 0.036779657 | 1.752021333 | Up |
| POS | 4-hydroxy-1-methyl-3-(phenylthio)-1,2-dihydroquinolin-2-one | 6.62 | 1.55565 | 0.01008994 | 2.572559195 | Up |
| POS | ethyl 3-oxo-3-(1H-pyrazol-5-ylamino)propanoate | 1.373 | 1.42273 | 0.042672512 | 1.308239628 | Up |
| POS | PC (18:4e/4:0) | 9.64 | 1.55858 | 0.006676838 | 2.144089697 | Up |
| POS | 3-methyl-5-oxo-5-(4-toluidino)pentanoic acid | 5.29 | 1.57972 | 0.001682073 | 1.573455132 | Up |
| POS | Oxymatrine | 6.823 | 1.54298 | 0.014672193 | 1.862919369 | Up |
| NEG | Androsterone | 9.563 | 1.61969 | 0.011487446 | 0.696508622 | Down |
| NEG | diethyl 2-[(4-methoxy-2-nitroanilino)methylidene]malonate | 5.105 | 1.53565 | 0.031641452 | 0.606362294 | Down |
| NEG | cis-5,8,11,14,17-Eicosapentaenoic acid | 9.384 | 1.65418 | 0.004245511 | 0.643131818 | Down |
| NEG | Gluconic acid | 1.359 | 1.5638 | 0.035125219 | 0.600552354 | Down |
| NEG | 2'-Deoxyinosine | 4.95 | 1.41902 | 0.04197093 | 0.419981077 | Down |
| NEG | LPE 22:4 | 9.573 | 1.62083 | 0.012793139 | 1.339508721 | Up |
| NEG | Chenodeoxycholic Acid | 7.136 | 1.64986 | 0.005748687 | 1.676759793 | Up |
| NEG | D-(+)-Galactose | 1.313 | 1.5505 | 0.02786938 | 1.280132688 | Up |
| NEG | Gamma-Glu-Leu | 4.962 | 1.50355 | 0.038382124 | 1.471543657 | Up |
| NEG | 16-Hydroxyhexadecanoic acid | 7.884 | 1.57692 | 0.020636128 | 1.322536928 | Up |
| NEG | Docosapentaenoic acid | 9.112 | 1.60256 | 0.008012228 | 1.201663431 | Up |
| NEG | N-METHYL (-)EPHEDRINE | 6.412 | 1.64573 | 0.004350214 | 1.807448635 | Up |
| NEG | N-Oleoyl Glycine | 9.644 | 1.53925 | 0.036338958 | 1.401561154 | Up |
| NEG | LPC 22:4 | 9.584 | 1.64207 | 0.004571093 | 1.41704615 | Up |

Table 2. Differential metabolites in group A compared to group CK

| Ion pattern | Peak | rt | VIP | Pvalue | FoldChange | significant |
| --- | --- | --- | --- | --- | --- | --- |
| POS | 2-(Formylamino)Benzoic Acid | 1.324 | 1.53567 | 0.009580234 | 0.738634206 | Down |
| POS | SM (d23:1/18:1) | 10.24 | 1.58838 | 0.0037864 | 0.70743134 | Down |
| POS | gamma-Glutamyltyrosine | 5.004 | 1.45638 | 0.035463049 | 0.794787824 | Down |
| POS | Isorhapontigenin | 5.735 | 1.39921 | 0.049036527 | 0.670475888 | Down |
| POS | SM (d29:0/15:0) | 10.246 | 1.49742 | 0.031809025 | 0.730484706 | Down |
| POS | 7-(2-aminophenyl)heptanoic acid | 6.611 | 1.48679 | 0.041259817 | 0.355969613 | Down |
| POS | 1-(4-benzylpiperazino)-2-(pyridin-2-ylamino)propan-1-one | 5.292 | 1.38461 | 0.046581016 | 0.482505819 | Down |
| POS | 2-(tert-butyl)-6,7-dimethoxy-4H-3,1-benzoxazin-4-one | 4.993 | 1.51791 | 0.009514616 | 0.51267725 | Down |
| POS | Kinetin | 5.023 | 1.4755 | 0.041294008 | 0.773595288 | Down |
| POS | p-Hydroxybenzaldehyde | 10.348 | 1.54427 | 0.009802338 | 0.711310275 | Down |
| POS | PC (18:2e/24:4) | 9.391 | 1.52969 | 0.048046382 | 0.566188124 | Down |
| POS | L-(-)-alpha-Amino-epsilon-Caprolactam | 9.641 | 1.40898 | 0.029311874 | 0.848771932 | Down |
| POS | Triptolide | 9.419 | 1.54019 | 0.041726744 | 0.209942345 | Down |
| POS | 4-(9H-fluoren-9-yl)pyridine | 4.906 | 1.54852 | 0.015056853 | 0.562027946 | Down |
| POS | Methamphetamine | 5.51 | 1.46591 | 0.025274661 | 0.815557798 | Down |
| POS | Stearoyl Ethanolamide | 6.84 | 1.49442 | 0.028000605 | 1.483146376 | Up |
| POS | 6,7,8-trimethoxy-2-(2-phenoxy-3-pyridyl)-4H-3,1-benzoxazin-4-one | 9.655 | 1.4217 | 0.021919638 | 1.392801818 | Up |
| POS | ACar 20:1 | 8.637 | 1.47004 | 0.032928285 | 1.262845057 | Up |
| POS | Biliverdin | 6.183 | 1.42465 | 0.024016304 | 1.538535113 | Up |
| POS | 3-Ureidopropionic acid | 1.476 | 1.59744 | 0.007504382 | 1.328282777 | Up |
| POS | ACar 10:1 | 5.991 | 1.43454 | 0.049129857 | 1.336894906 | Up |
| POS | ACar 10:2 | 5.832 | 1.3423 | 0.044543534 | 1.404212781 | Up |
| POS | N-Methylhydantoin | 1.458 | 1.45853 | 0.038495965 | 1.330894272 | Up |
| POS | ACar 20:2 | 8.207 | 1.49989 | 0.027916037 | 1.353698806 | Up |
| POS | ACar 22:6 | 7.512 | 1.54651 | 0.031838678 | 1.485909502 | Up |
| POS | Palmitoylcarnitine | 7.811 | 1.38385 | 0.043458026 | 1.246595835 | Up |
| POS | Methoxyacetyl fentanyl-d5 | 6.076 | 1.61221 | 0.003278337 | 1.512272971 | Up |
| POS | N-Isovalerylglycine | 5.368 | 1.44483 | 0.03197116 | 1.327604405 | Up |
| POS | Proline | 1.428 | 1.4501 | 0.037722326 | 1.100419837 | Up |
| POS | D-Glucose 6-phosphate | 1.351 | 1.56577 | 0.008424718 | 1.464684721 | Up |
| POS | N6,N6,N6-Trimethyl-L-lysine | 1.284 | 1.58736 | 0.022172071 | 1.30936068 | Up |
| POS | Linoleoyl ethanolamide | 9.36 | 1.47548 | 0.024238757 | 1.454061426 | Up |
| POS | DL-Î±-Aminocaprylic acid | 5.584 | 1.47615 | 0.049820154 | 1.207163278 | Up |
| POS | (-)-Caryophyllene oxide | 1.354 | 1.42677 | 0.041884145 | 1.248405607 | Up |
| POS | Thymidine | 4.91 | 1.45492 | 0.036008011 | 1.150245456 | Up |
| POS | DL-Arginine | 1.304 | 1.55093 | 0.032417154 | 1.280202954 | Up |
| POS | Guanidineacetic acid | 1.36 | 1.47176 | 0.00828196 | 1.31914463 | Up |
| POS | ACar 17:1 | 7.66 | 1.48998 | 0.021761121 | 1.348325485 | Up |
| POS | 3-(2-Naphthyl)-D-Alanine | 6.312 | 1.40145 | 0.039980499 | 1.593097896 | Up |
| POS | PC (18:5e/4:0) | 9.12 | 1.39341 | 0.036716482 | 1.395213663 | Up |
| POS | Tetrahydrocortisone | 6.096 | 1.61509 | 2.79783E-05 | 1.324402707 | Up |
| POS | Nicotinamide | 2.056 | 1.45604 | 0.032324849 | 1.226828851 | Up |
| POS | PC (20:2/20:3) | 10.639 | 1.53081 | 0.020607289 | 16.12653669 | Up |
| POS | PC (18:3e/2:0) | 9.276 | 1.49159 | 0.036779657 | 1.752021333 | Up |
| POS | 4-hydroxy-1-methyl-3-(phenylthio)-1,2-dihydroquinolin-2-one | 6.62 | 1.55565 | 0.01008994 | 2.572559195 | Up |
| POS | ethyl 3-oxo-3-(1H-pyrazol-5-ylamino)propanoate | 1.373 | 1.42273 | 0.042672512 | 1.308239628 | Up |
| POS | PC (18:4e/4:0) | 9.64 | 1.55858 | 0.006676838 | 2.144089697 | Up |
| POS | 3-methyl-5-oxo-5-(4-toluidino)pentanoic acid | 5.29 | 1.57972 | 0.001682073 | 1.573455132 | Up |
| POS | Oxymatrine | 6.823 | 1.54298 | 0.014672193 | 1.862919369 | Up |
| NEG | Androsterone | 9.563 | 1.61969 | 0.011487446 | 0.696508622 | Down |
| NEG | diethyl 2-[(4-methoxy-2-nitroanilino)methylidene]malonate | 5.105 | 1.53565 | 0.031641452 | 0.606362294 | Down |
| NEG | cis-5,8,11,14,17-Eicosapentaenoic acid | 9.384 | 1.65418 | 0.004245511 | 0.643131818 | Down |
| NEG | Gluconic acid | 1.359 | 1.5638 | 0.035125219 | 0.600552354 | Down |
| NEG | 2'-Deoxyinosine | 4.95 | 1.41902 | 0.04197093 | 0.419981077 | Down |
| NEG | LPE 22:4 | 9.573 | 1.62083 | 0.012793139 | 1.339508721 | Up |
| NEG | Chenodeoxycholic Acid | 7.136 | 1.64986 | 0.005748687 | 1.676759793 | Up |
| NEG | D-(+)-Galactose | 1.313 | 1.5505 | 0.02786938 | 1.280132688 | Up |
| NEG | Gamma-Glu-Leu | 4.962 | 1.50355 | 0.038382124 | 1.471543657 | Up |
| NEG | 16-Hydroxyhexadecanoic acid | 7.884 | 1.57692 | 0.020636128 | 1.322536928 | Up |
| NEG | Docosapentaenoic acid | 9.112 | 1.60256 | 0.008012228 | 1.201663431 | Up |
| NEG | N-METHYL (-)EPHEDRINE | 6.412 | 1.64573 | 0.004350214 | 1.807448635 | Up |
| NEG | N-Oleoyl Glycine | 9.644 | 1.53925 | 0.036338958 | 1.401561154 | Up |
| NEG | LPC 22:4 | 9.584 | 1.64207 | 0.004571093 | 1.41704615 | Up |

## 2. Supplementary Figures


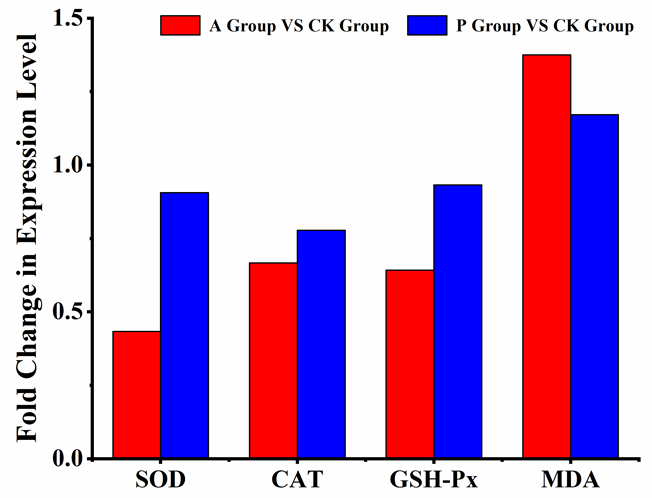


**Figure S1.** Statistical comparison of SOD, CAT, and MDA expression levels in different groups.


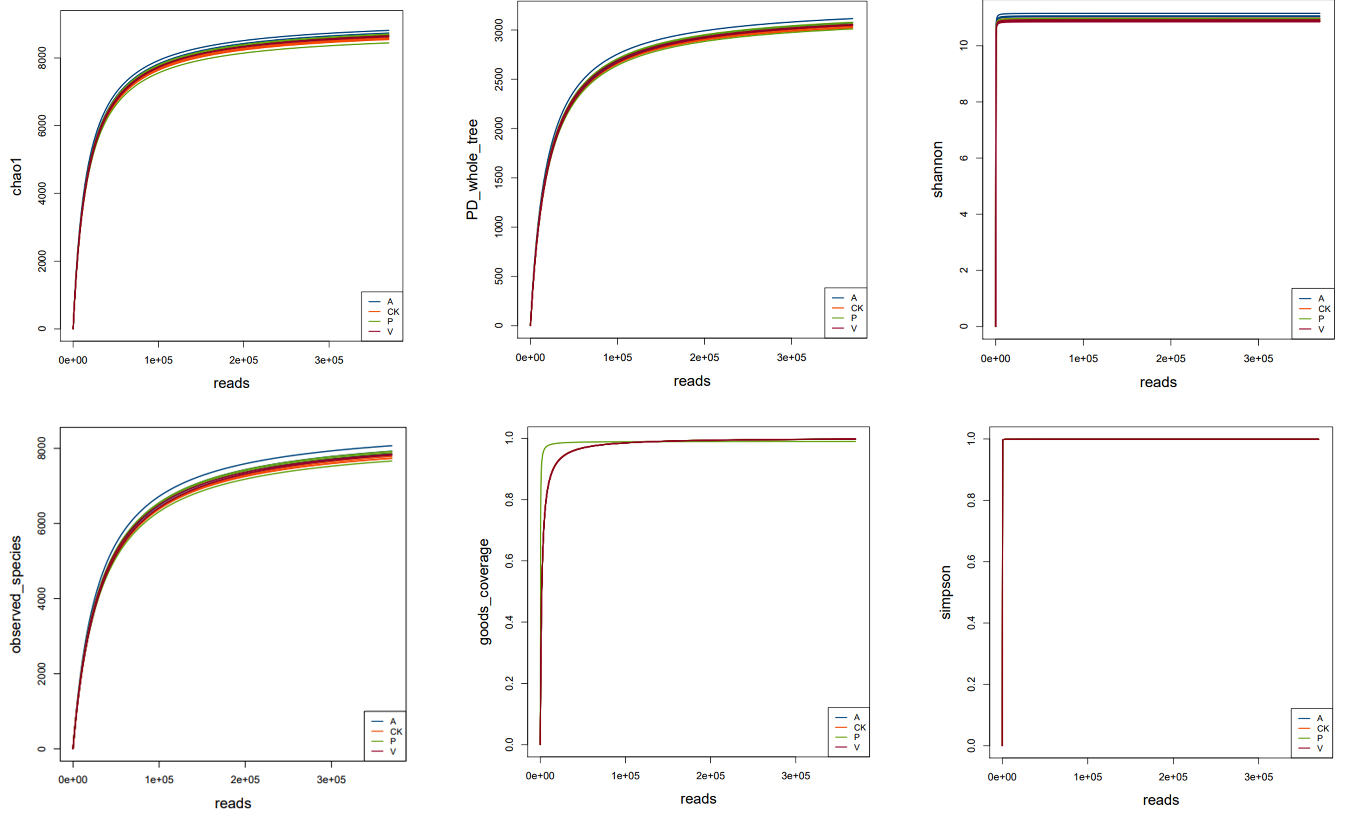


**Figure S2.** Alpha diversity analysis - dilution curve.


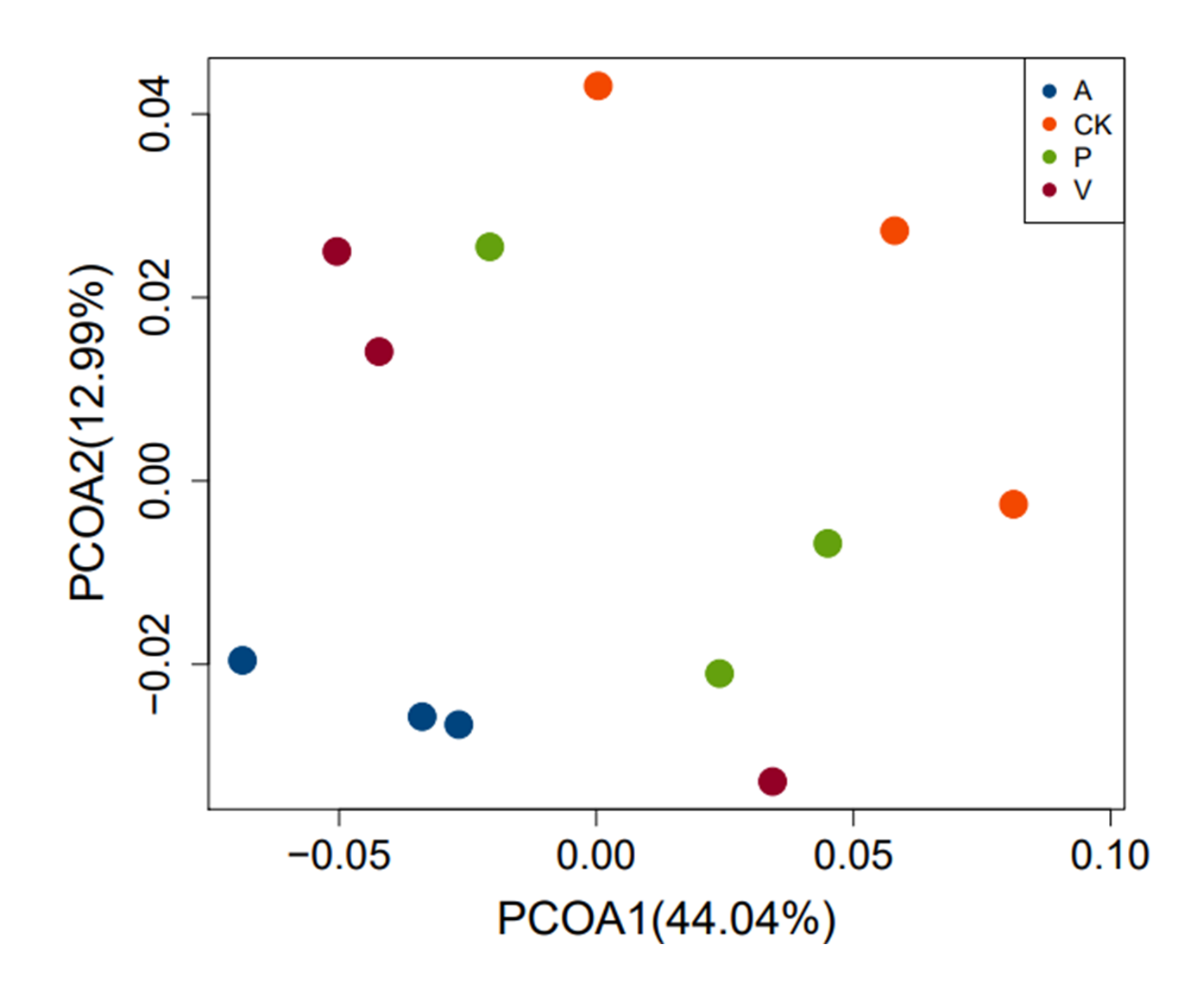


**Figure S3.** Beta diversity analysis.
